# Huge epithelioid malignant peripheral nerve sheath tumor in the left axilla: a case report

**DOI:** 10.1186/s40792-015-0065-x

**Published:** 2015-08-12

**Authors:** Eiji Kusumoto, Shohei Yamaguchi, Masahiko Sugiyama, Mitsuhiko Ota, Norifumi Tsutsumi, Yasue Kimura, Yoshihisa Sakaguchi, Tetsuya Kusumoto, Koji Ikejiri, Yoshifuku Nakayama, Seiya Momosaki

**Affiliations:** Department of Gastroenterological Surgery, Clinical Research Center Cancer Research Division, National Hospital Organization Kyushu Medical Center, 1-8-1, Jigyohama, Chuo-ward, Fukuoka-city, Fukuoka 810-8563 Japan; Department of Pathology, Kyushu Medical Center, 1-8-1, Jigyohama, Chuo-ward, Fukuoka-city, Fukuoka 810-8563 Japan; Department of Gastroenterological Surgery, National Kyushu Medical Center 1-8-1, Jigyohama, Chuo-ward, Fukuoka, 810-8563 Japan

**Keywords:** Epithelioid malignant peripheral nerve sheath tumor, Extended resection, Chromosomally unstable

## Abstract

This report describes a patient with a rare huge epithelioid malignant peripheral nerve sheath tumor (MPNST) in the left axilla. A male in his 70s was admitted to our hospital for evaluation of a growing tumor in his left axilla. The tumor was solid and immovable. Examination of a biopsy specimen resulted in a diagnosis of epithelioid MPNST. Two weeks after the biopsy was performed, the tumor grew to 20 cm and became painful, and the patient was unable to feel pressure on his upper arm. Immediately before surgery to remove the tumor, computed tomography suggested the presence of lung metastases. The patient and his family were informed of his disease state, and they elected surgical treatment to ease the symptoms associated with tumor enlargement. Systemic metastases appeared soon after the surgery, and the patient died within 11 weeks. Comparative genomic hybridization (CGH) analysis showed that this tumor was chromosomally unstable, with impairments in gene expression.

## Background

Malignant peripheral nerve sheath tumors (MPNSTs), which originate from peripheral nerve cells, account for approximately 3 to 10 % of malignant soft tissue tumors [1]. A histological subtype, epithelioid MPNST, involving epithelioid tumor cells, is uncommon, accounting for 5 to 17 % of MPNSTs [2]. The first line and only treatment is extended resection.

This report describes a rare huge epithelioid MPNST in the left axilla of a male in his 70s. Extended resection could not be performed because of lung metastases. Systemic metastasis appeared soon after surgery, and the patient died within 11 weeks postoperatively. This report describes our experience with this patient and presents a review of the literature.

## Case presentation

Patient: Male in his 70s.

Chief complaint: Growing tumor in the left axilla.

Past history: None.

History of present illness: The patient felt a growing tumor in his left axilla, for which he was admitted to our hospital.

**Fig. 1:**
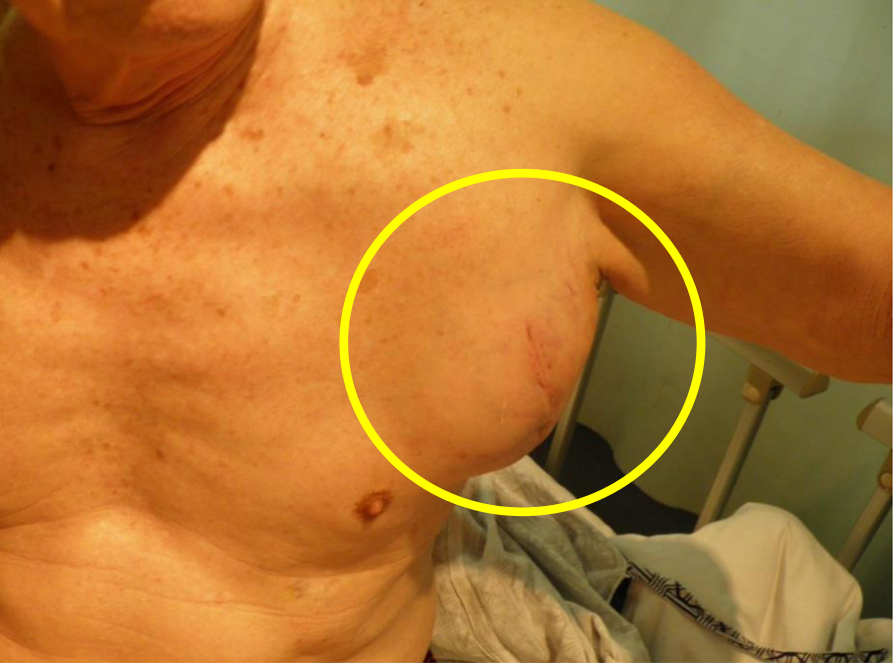
Physical findings on initial examination. The tumor in the left axilla was fist-sized, solid, and immovable. A scar from a biopsy incision overlay the tumor

**Fig. 2:**
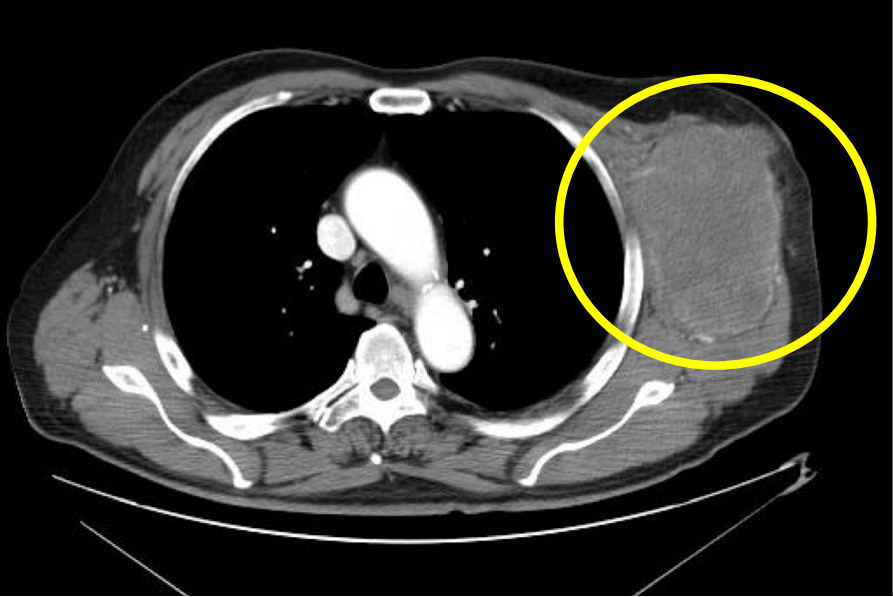
Contrast-enhanced chest computed tomography. The tumor in the left axilla measured 11 cm × 7 cm × 11 cm. It had a relatively distinct border, but its surface was irregular

Examinations: Physical findings on initial examination included an elastic, hard, fist-sized tumor in the left axilla; the tumor was solid and immovable (Fig. 1). Ultrasound examination showed that the tumor was well-circumscribed and lobulated, with a maximum diameter of 12 cm. The inner portion of the tumor was uneven with mixed echogenicity; areas of strong echogenicity indicated abundant blood flow. Contrast-enhanced chest computed tomography showed a tumor measuring 11 cm × 7 cm × 11 cm, with relatively distinct borders, but irregular surfaces (Fig. 2). No findings suggestive of liver or lung metastases were observed.

**Fig. 3:**
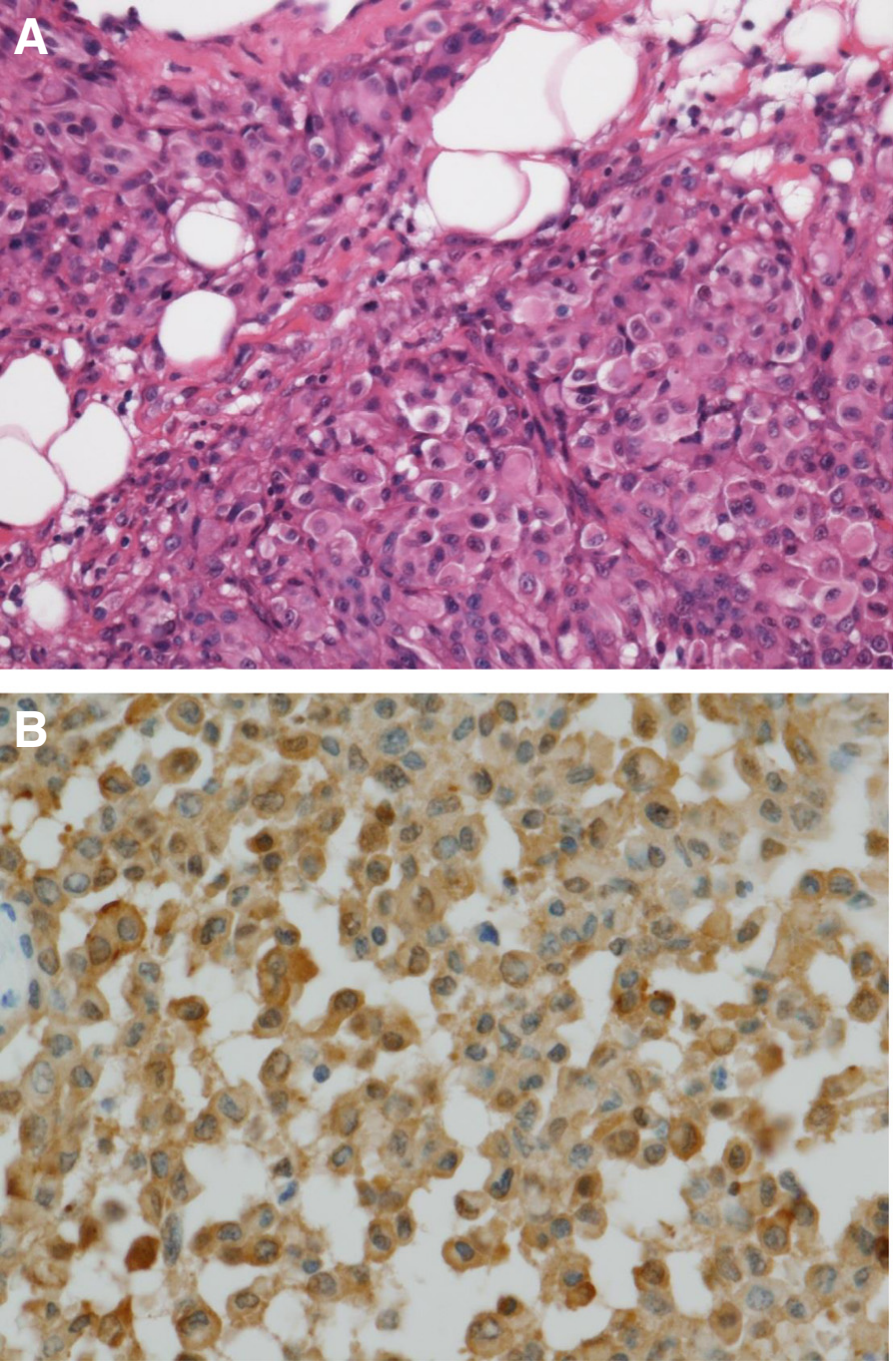
Histopathological biopsy findings. **a** Hematoxylin-and-eosin staining (high-power field). Atypical epithelial cells with spindle-shaped nuclei and rich cytoplasm were present in dense sheets. **b** S-100 staining. The tumor was strongly and diffusely positive for S-100 protein

Histology: Histopathological examination of a biopsy specimen showed densely sheathed atypical epithelial cells with spindle-shaped nuclei and rich cytoplasm (Fig. 3a). The tumor was strongly and diffusely positive for vimentin, S-100 protein (Fig. 3b), and neuron-specific enolase, a pattern of immunoreactivity differing from that of conventional MPNST. However, the tumor was negative for epithelial membrane antigen, cytokeratin AE1/AE3, c-kit, CD34, α-smooth muscle actin, desmin, synaptophysin, HMB-45, and calretinin, indicating that metastatic cancer, malignant melanoma, and clear cell sarcoma were unlikely. The tumor was diagnosed as an epithelioid MPNST.

Progress after hospitalization: Two weeks after the biopsy, the tumor in the left axilla grew to 20 cm in diameter. It became very painful, and the patient was unable to feel pressure on his upper arm. Computed tomography immediately before surgery suggested the presence of minute lung metastases. The patient and his family were informed of his disease state, and they elected surgical treatment to ease the symptoms associated with tumor enlargement.

Surgical findings: A combination of general and epidural analgesia was administered with the patient lying on his right side. A T-shaped incision was made directly above the tumor in the left axilla. The tumor had a maximum diameter of 21 cm, was relatively inflexible, and had invaded the muscles of the thoracic wall; however, it had not invaded the ribs. Many swollen lymph nodes, located from the interpectoral area (Rotter’s lymph node) to the left supraclavicular fossa, were connected to the main tumor. We removed as many lymph nodes as possible to ease the symptoms associated with tumor enlargement.

Pathology of the tumor: The tumor measured 21 cm × 17 cm × 8 cm in size and weighed 905 g. Tumor cells had infiltrated the trapezius muscle and thoracic wall, accompanied by axillary (7/8) and interpectoral (6/6) lymph node metastases. The tumor contained an epithelioid portion with invasive growth of polymorphic cells, including morphologically abnormal and oval-to-round cells, intermixed with the sarcoma portion, consisting of spindle cells and associated necrosis.

Postoperative progress: The patient’s pain resolved after surgery. Two weeks later, however, the minute lung metastases had grown larger. Five weeks later, pleural and peritoneal dissemination and bone marrow metastases were observed. Eight weeks later, the patient began to experience respiratory discomfort, and his overall condition worsened. He died within 11 weeks postoperatively.

**Fig. 4:**
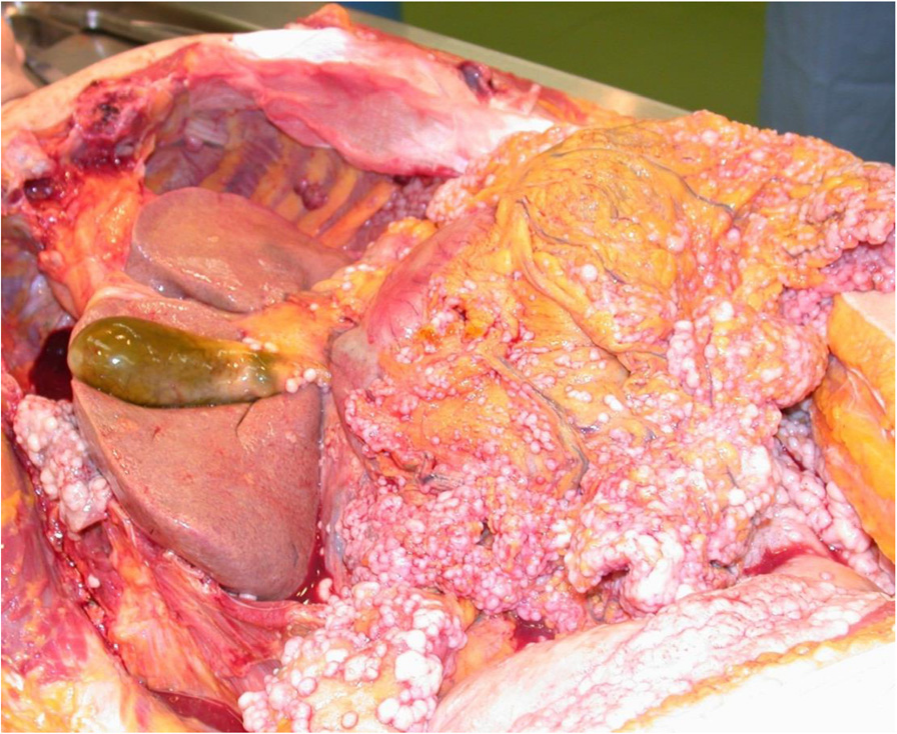
Pathological autopsy. Metastatic tumor cells were found in the peritoneum, lymph nodes, pharynx, pleura, lungs, pericardium, endocardium, myocardium, liver, omentum, pancreas, splenic hilum, stomach, jejunum, ileum, transverse colon, appendix, and bone marrow. Informed consent to publish this photograph was obtained from the patient before death and from the patient’s family after death

Pathology at autopsy (Fig. 4): An autopsy was performed 24 h after death. Other than extensive pleural and peritoneal dissemination, various metastatic lesions were observed throughout both lungs (left, 515 g; right, 690 g) and both liver lobes (1380 g). A total of 4500 ml of ascites fluid had accumulated, and microscopic examination showed metastatic tumor cells in the peritoneum, including in the rectovesical pouch, lymph nodes (cervical, axillary, interpectoral, peritracheal, peribronchial, para-aortic, and cystic duct), pharynx, pleura, both lungs, pericardium, endocardium, myocardium (415 g), liver, omentum, pancreas, splenic hilum, stomach, jejunum, ileum, transverse colon, appendix, and bone marrow.

## Discussion

Prognostic factors in patients with MPNST include neurofibromatosis-1 (NF1) complications, tumor diameter, site of onset, and excisional margins. NF1 complications are found in roughly half of patients with MPNST but in fewer than 2 % of patients with epithelioid MPNST [1, 2]. Survival appears to be lower in patients with NF-1-associated MPNST than in patients with sporadic MPNST. Local recurrence and distant metastases are frequent in patients with tumors >5 cm in diameter and are associated with poor outcome [3]. Frequency of onset is similar in the limbs and trunk, although onset in deep tissues has been associated with poor prognosis. No differences between males and females have been observed [4–6]. Maintenance of excisional margins at the time of surgery has been associated with a significant reduction or prevention of local recurrence [5]. Although the optimal excisional margins have not been established, extended resection is the treatment of choice for malignant soft tissue tumors [2, 4, 5]. The efficacy of radiotherapy and chemotherapy has not yet been established.

The prognosis of genetic findings in patients with epithelioid MPNST remains unclear because of the small number of patients and the variation in survival rates [6, 7]. Autopsy findings in this patient showed that systemic metastases appeared soon after surgery. Microscopic examination showed metastatic tumor cells in the peritoneum, lymph nodes, pharynx, pleura, both lungs, pericardium, endocardium, myocardium, liver, omentum, pancreas, splenic hilum, stomach, jejunum, ileum, transverse colon, appendix, and bone marrow. Lymphogenous metastasis is the main metastatic pattern, whereas metastasis to the gastrointestinal tract is generally disseminated.

**Fig. 5:**
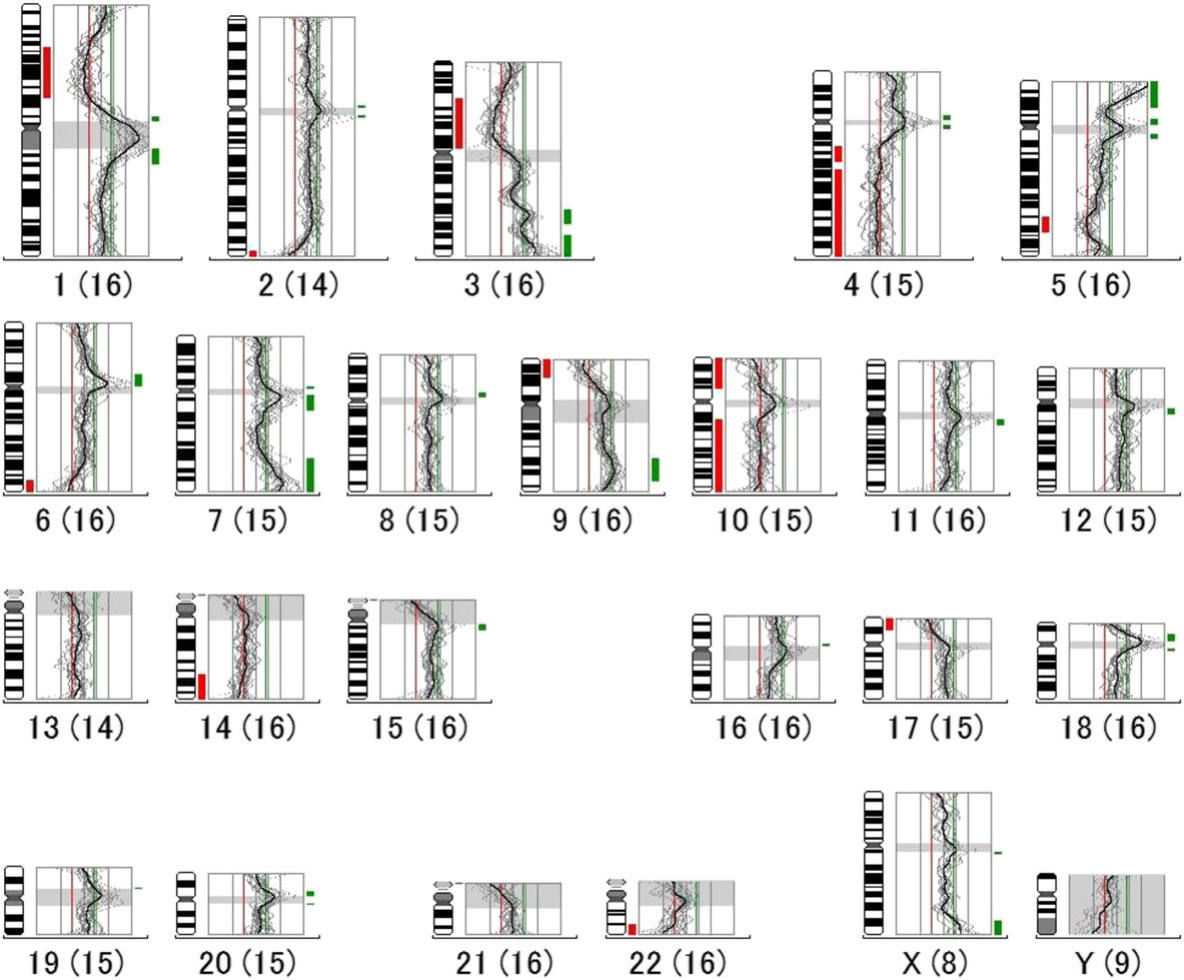
CGH analysis of chromosomal abnormalities. Losses in DNA copy number were observed at chromosomes 1p, 3p, 4q, 5q, 6p, 9p, 10p, 14q, 17p, and 22p and gains in DNA copy number at chromosomes 3q, 5p, 7q, 9p, and Xp

The differential diagnoses of epithelioid MPNST include metastatic cancer, malignant melanoma, and clear cell sarcoma. The diagnosis of epithelioid MPNST should be determined by histopathological examination, including immunohistochemistry [8, 9], and CGH analysis of chromosomal abnormalities [3]. Our patient showed losses in DNA copy numbers on chromosomes 1p, 3p, 4q, 5q, 6p, 9p, 10p, 14q, 17p, and 22p and gains in DNA copy numbers on chromosomes 3q, 5p, 7q, 9p, and Xp (Fig. 5). These findings suggest that the epithelioid MPNST in our patient may have been chromosomally unstable, with impairments in gene expression.

## Conclusions

We report here a patient with a giant epithelioid MPNST in the left axilla. The patient underwent surgery to ease the symptoms associated with tumor enlargement. Systemic metastases appeared soon after the surgery, and the patient died within 11 weeks.

## Consent

Informed consent was obtained from the patient’s family for publication of this case report and any accompanying images. A copy of the written consent is available for review by the Editor-in-Chief of this journal.

## Abbreviations

CGH: comparative genomic hybridization
MPNST: malignant peripheral nerve sheath tumor
NF1: neurofibromatosis-1
